# Prolonged Internal Displacement and Common Mental Disorders in Sri Lanka: The COMRAID Study

**DOI:** 10.1371/journal.pone.0064742

**Published:** 2013-05-22

**Authors:** Chesmal Siriwardhana, Anushka Adikari, Gayani Pannala, Sisira Siribaddana, Melanie Abas, Athula Sumathipala, Robert Stewart

**Affiliations:** 1 Health Service and Population Research Department, Institute of Psychiatry, King's College London, London, United Kingdom; 2 Institute for Research and Development, Colombo, Sri Lanka; 3 Department of Medicine, Faculty of Medicine and Allied Sciences, Rajarata University, Anuradhapura, Sri Lanka; Tabriz University of Medical Sciences, Iran (Islamic Republic of)

## Abstract

**Background:**

Evidence is lacking on the mental health issues of internally displaced persons, particularly where displacement is prolonged. The COMRAID study was carried out in year 2011 as a comprehensive evaluation of Muslims in North-Western Sri Lanka who had been displaced since 1990 due to conflict, to investigate the prevalence and correlates of common mental disorders.

**Methods:**

A cross-sectional survey was carried out among a randomly selected sample of internally displaced people who had migrated within last 20 years or were born in displacement. The total sample consisted of 450 adults aged 18–65 years selected from 141 settlements. Common mental disorders (CMDs) and post-traumatic stress disorder (PTSD) prevalences were measured using the Patient Health Questionnaire and CIDI sub-scale respectively.

**Results:**

The prevalence of any CMD was 18.8%, and prevalence for subtypes was as follows: somatoform disorder 14.0%, anxiety disorder 1.3%, major depression 5.1%, other depressive syndromes 7.3%. PTSD prevalence was 2.4%. The following factors were significantly associated with CMDs: unemployment (odds ratio 2.8, 95% confidence interval 1.6–4.9), widowed or divorced status (4.9, 2.3–10.1) and food insecurity (1.7, 1.0–2.9).

**Conclusions:**

This is the first study investigating the mental health impact of prolonged forced displacement in post-conflict Sri Lanka. Findings add new insight in to mental health issues faced by internally displaced persons in Sri Lanka and globally, highlighting the need to explore broader mental health issues of vulnerable populations affected by forced displacement.

## Introduction

Internal conflicts causing forced displacement of non-combatant populations are a common global occurrence [1], and usually associated with substantial health and social impacts on internally displaced persons (IDPs) including acute and long-term effects on mental health [2]–[4]. Mental health issues related to forced displacement have been extensively studied in conflict-affected refugee and IDP populations [5]–[8]. However, limited evidence is available about the impact of prolonged internal displacement [9].

Forced displacement can last from short intervals (a few months) to much longer periods (decades or over several generations) [10]. The nature of the conflict that caused displacement, the ongoing geo-political situation and the choices of the displaced population may define the outcome of the displacement process/post-flight phase [10]. Unlike refugees, IDPs may not have a choice in ending displacement as they are displaced within national boundaries, often under the control of parties responsible for displacement and not covered by international legal conventions applying to refugees [11]–[13]. IDPs are often neglected when displacement is prolonged, with adverse social, cultural, economical and health impacts [14]–[16]. Continuation of conflict in the area of origin has been found to be associated with poorer mental health outcomes in those displaced [17], [18]. Most forced and prolonged displacement takes place against a backdrop of resource-poor settings where social vulnerability, lack of adequate infrastructure along with loss of hope for the future can act to compound the already raised risk of mental disorders among IDP populations [18].

Mental disorders associated with forced internal displacement are varied [3], and most studies have focused on a limited numbers of disorders, such as PTSD, anxiety and depression [19]. Current epidemiological research focusing on the broader common mental disorder (CMD) spectrum related to forced displacement is limited [9], [19]. Within the limited available global evidence, CMD prevalence is seen to vary substantially: 27.2% in Colombia, 27.8% in Ethiopia, 40.3% in Palestine, 57.7% in Cambodia, 62.3% in Algeria [19]–[22]. In addition, these studies were conducted among a mix of refugee and IDP populations and do not provide clear information about the duration of displacement periods, making it difficult to clearly assess the mental health impact of prolonged displacement. The dearth of an epidemiological evidence-base inhibits the formulation of effective interventions, despite the fact that prolonged internal displacement presents important yet complex public health challenges to resource-poor affected nations, as well as to the numerous international agencies involved [7], [23], [24].

Against this backdrop, the ‘COmmon Mental Disorders and Resilience Among Internally Displaced in Sri Lanka (COMRAID)’ study aimed to describe the prevalence of CMD (including depression, anxiety and somatoform disorders), and post traumatic stress disorder among adult Muslims affected by conflict-driven prolonged displacement since 1990 in the Puttalam district of north-western Sri Lanka. Although there has been some research into trauma and mental disorders among people affected by the three decades of civil conflict in Sri Lanka [22], COMRAID is the first comprehensive study to explore prolonged forced displacement and its mental health impact in the country. This paper presents findings on the outcomes of CMD and PTSD prevalence and their associations with socio-demographic/socio-economic factors.

## Methodology

### Ethics statement

Informed written consent was obtained from each participant and an information leaflet provided, with a copy of the signed consent form. The ethical approval for the study was obtained from the Psychiatry, Nursing and Midwifery Research Ethics Subcommittee of King's College London and the Ethics Review Committee, Faculty of Medicine, University of Sri Jayewardenepura in Sri Lanka. Ethical challenges faced by the research team during the study have been discussed elsewhere in detail [25].

### Study design and participants

Sri Lanka, a multi-ethnic developing country with a mid-year population of 20 million estimated in 2005 and some of the best health and educational indicators in the South Asian region [26], has seen many instances of internal displacement, but these have been characteristically unpredictable and fluid, the main precipitants being the three decades of conflict and the 2004 Tsunami [27].

**Figure 1:**
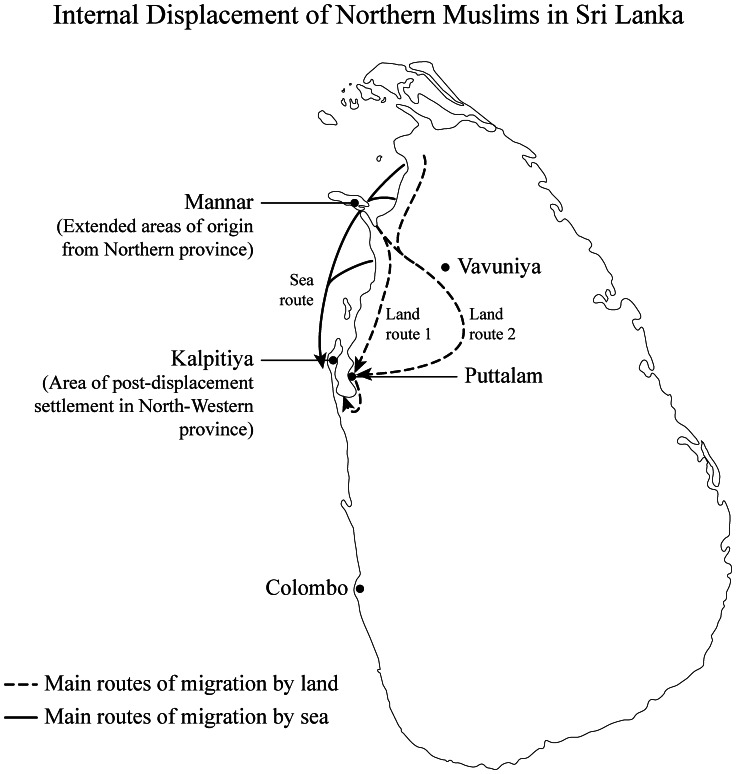
Internal Displacement of Northern Muslims in Sri Lanka. Areas of origin, displacement routes and post-displacement resettled areas are shown.

The COMRAID cross-sectional survey was carried out in the first half of 2011 in the Kalpitiya division of Puttalam district, North-western province of Sri Lanka. Puttalam district, which has long had a majority Muslim population [28], [29], has been an accessible safe haven for a large number of IDPs due to its geographical closeness to conflict areas. The Kalpitiya division (a small peninsular landmass separated from the mainland by a lagoon) was selected for sampling as it had the largest concentration of IDPs within a relatively small geographic area. It is estimated that around 75,000 Muslims (a distinct minority ethnic group in Sri Lanka, whose religion is Islam, and language mainly Tamil) were displaced over a period of few days in 1990 from the northern regions. The majority (72%) were displaced from Mannar district of the Northern Province [28]. According to official sources, Puttalam district received around 15,000 of these internally displaced families (63,000 individuals), mainly arriving via the sea route, who were initially resettled in temporary shelters in the peninsula in 141 locations (mainly welfare centres) [30], [31]. Figure 1 illustrates the displacement routes and resettlement areas of the northern Muslim IDPs.

Over a time span of 20 years in forced displacement, as the population numbers grew, a proportion of IDPs bought local land, establishing communities based on their pre-displacement communal orientations while others have lived in government welfare centres for the whole duration of displacement. Living circumstances and economic standards are similar across these different types of settlements, and some of the privately established communities in fact function as welfare centres. The 141 welfare camps in existence at the time of the survey were mostly situated in government land and had basic facilities, consisting of permanent, semi-permanent and temporary housing for IDP families numbering from less than hundred families to several hundred. Camps were overseen by a ‘camp officer’ (the village level administrative official) appointed by the government while civil society organizations such as mosque councils were also responsible for handling various communal affairs. The flight of northern Muslims has been well documented in sociological and anthropological studies [32]–[34]; however, no study to date has investigated the mental health status of these IDPs, considered to be at high risk for developing psychopathology due to the nature of their displacement experience.

### Sampling

The inclusion criteria used in the study were as follows: i) Sri Lankan nationality, ii) aged between 18 to 65 years, iii) previously resident in the Northern Province of Sri Lanka, iv) displaced in 1990 and residing in welfare camps and other settlements in Kalpitiya division of Puttalam district since, or born to displaced parents (at least one parent).

No prior prevalence data on mental disorders were available for the Northern Muslim IDPs living in the study area. However, prevalence data for Puttalam district was available from a national survey (major depression 4.2%, other depressive syndromes 11.9%, somatoform disorder 8.9%) [35]. A sample size of 158 was calculated as being required (alpha 0.05, 90% power), assuming a 10% prevalence of common mental disorders. As the study used a cluster sampling method with a design effect of 2.0, total sample size was doubled. Allowing for a 70% response rate, it was decided to approach 450 IDPs for participation, allowing precision (95% confidence interval) of 2% around a characteristic with 5% prevalence, and 3% around a characteristic with 10% prevalence for descriptive analyses [36].

A multi-stage sampling strategy was used. Welfare camps were randomly selected based on probability proportionate to the size of each camp population. In the first stage, 45 camps (for 450 participants) were selected according to the population of IDPs in each camp from the total of 141 welfare camps in the Kalpitiya division. The camp list and population statistics were obtained from the Northern Muslim Secretariat of the Ministry of Resettlement, Government of Sri Lanka. In the second stage, ten households were randomly selected from each selected welfare camp using a list of camp residents provided by the camp officer. Subsequently, each household was approached and an eligible member randomly selected using the Kish method [37].

### Study instruments and variables

The COMRAID study gathered information from the participants using a structured interview, consisting of several components. Demographic and economic characteristics, mental health outcomes (prevalence of common mental disorders, PTSD), current alcohol use, current drug use and current smoking prevalence were measured. Mental health outcomes were measured using the Primary Care Evaluation of Mental Disorders Patient Health Questionnaire (PRIME-MD PHQ) [38]. This has been widely applied in cross-cultural research [39] and is designed to diagnose the most common mental disorders (somatoform disorders, major depression, other depressive syndromes, and anxiety) according to DSM-IV criteria. Post-traumatic stress disorder (PTSD) was separately measured using the K-section of the Composite International Diagnostic Interview (CIDI) and socio-demographic information was gathered using a standard questionnaire [39]. The majority of instruments had been previously used in large-scale epidemiological studies in Sri Lanka [35], [40], and had been translated, back translated and evaluated for cross-cultural equivalence [41]. The full questionnaire was piloted in a volunteer sample in the preparatory phase of the COMRAID study to check applicability. Input from key informants of the IDP community was used to refine questionnaire items. Tamil language versions (Cronbach alpha scores 0.861 for the PHQ, 0.755 for the CIDI K) of the questionnaire were used, as the main language spoken among the Muslim IDPs, and Sinhala and English language versions were also available if required.

Taking into account current age, the sample was categorised into the following three groups: i) born after displacement in 1990, ii) child at displacement in 1990, and iii) adult (age 18+) at displacement in 1990. Education level was coded according to the existing Sri Lankan categorisation of primary (up to grade 5), secondary (up to ordinary level - OL – grade 11) and post-secondary (advance level and above - AL – grade 13). Food insecurity was measured by the reported total number of days without sufficient food to meet the needs of the household of a participant, covering the calendar year before the date of the interview, and was categorised as a binary entity on the basis of more than 60 such days per year. Participants were also asked about current debt. If a participant was without permanent, part-time or casual work at the time of the interview, they were considered as unemployed.

### Data collection and data entry

Survey data was collected by a team of research assistants, recruited and trained for the study. To avoid bias, these were recruited from areas of Puttalam and Mannar districts without direct contact to the IDP communities. The team comprised of 5 females (2 of Muslim ethnicity and 3 of Tamil ethnicity) and 1 male (Muslim ethnicity) with prior field experience in social research. Two were university graduates while the others had advanced level (post-secondary) education. All were native Tamil language speakers and were conversant in Sinhala and English. A gender-matched data collection strategy was used to account for community sensitivities. All interviewers were provided with initial and refresher training sessions by the academic study team comprising of psychiatrists, epidemiologists and bioethicists on basic mental health knowledge, on the specific nature of the study and instruments, and on ethical aspects of research (informed consent, confidentiality). Supervision was provided by the PI together with a medically trained project coordinator, also responsible for handling participant queries and the extended IDP community. Double data entry was performed by 4 operators, supervised by a statistician, using SPSS software [42].

### Data analysis

Data analyses were conducted using STATA version 10 [43]. All analyses were adjusted for cluster sampling design and all results are presented as weighted data. The prevalence of mental disorders and associations between mental disorders and socio-demographic/socio-economic variables was investigated through descriptive analysis, followed by unadjusted regression analyses of associations between any CMD and relevant socio-demographic/socio economic variables. Correlational analysis using Pearson product-moment correlation coefficient found significant co-linearity between unemployment and variables of gender (r = 0.749, n = 450, p<0.01) and education (r = 0.206, n = 450, p<0.01). Therefore, we explored two separate models in logistic regression analysis of associations between CMD and demographic/economic variables. The first model included CMD (any CMD/sub groups), age at displacement, gender, marital status, education, financial debt and food security variables. The second model included CMD (any CMD/sub groups), age at displacement, gender, marital status, education, financial debt, food security and employment.

## Results

### Socio-demographic profile

A total of 450 participants were interviewed with an overall 100% response rate obtained. The appropriately weighted distributions of covariates in the sample are displayed in Table 1. The mean age was 37.1 years (standard error 0.57), female representation was high, with sizeable numbers reporting financial indebtedness and/or food insecurity during the past year.

**Table 1:** Socio-demographic characteristics and CMD prevalence.

			CMD sub-group				
	Number in group	Any CMD	Somato-form disorder	Major Depression	Other Depression	Anxiety	PTSD
Characteristic	N = 450	%	%	%	%	%	%
**Age**							
*18–21 (born after displacement)*	39	10.2 (0.7–9.7)	5.1	2.5	5.1	0	0
*22–37 (child at displacement)*	189	11.1 (6.6–5.5)	9.5	3.7	2.1	1.5	2.1
*38–65 (adult at displacement)*	222	27.0 (21.1–32.8)	19.3	6.7	12.1	1.3	3.1
**Gender**							
*Male*	166	14.4 (9.0–19.7)	9.0	4.8	6.6	1.8	1.2
*Female*	284	21.4 (16.6–26.1)	16.9	5.2	7.7	1.0	3.1
**Marital status**							
*Married*	345	17.6 (13.5–21.6)	14.2	3.7	6.6	1.1	1.7
*Widow/Divorced*	37	51.3 (35.1–67.4)	27.0	21.6	21.6	0	8.1
*Never married*	67	7.4 (1.1– 13.6)	5.9	2.9	2.9	2.9	2.9
**Employment**							
*Employed*	182	10.4 (5.9–14.8)	7.1	3.2	4.3	0.5	2.1
*Unemployed*	268	24.6 (19.4–29.7)	18.6	6.3	9.3	1.8	2.6
**Education**							
*Primary (Up to grade 5)*	115	30.4 (21.9–38.8)	20.9	9.6	13	0.9	2.6
*Secondary (Up to OL)*	272	16.2 (11.8–20.5)	12.5	4.7	5.9	1.5	2.6
*Post-secondary (AL & above)*	61	9.8 (2.3–17.2)	8.2	1.6	3.3	1.6	1.6
**Ethnicity**							
*Muslim*	424	19.6 (15.8–23.3)	14.3	5.1	7.5	1.4	2.5
*Other*	24	8.3 (−2.7–19.3)	8.3	4.1	4.1	0	0
**Financial debt**							
*Indebted*	193	18.7 (13.2–24.2)	14.0	4.7	9.3	1.6	2.6
*No debts*	256	18.8 (14.0–23.5)	13.7	5.5	5.9	1.2	2.3
**Food security(last year)**							
*Sufficient food*	319	16.3 (12.2–20.3)	13.1	4.0	4.9	0.3	1.8
*Lack of sufficient food*	130	25.4 (17.9–32.8)	16.2	7.7	13.8	3.8	3.8

### Prevalence of common mental disorders (CMD)

The prevalence of any common mental disorder among the study population was 18.8% (95%CI 15.2–22.5). Somatoform disorder (14.0%, 95%CI 10.7–17.9) was the most common sub-category followed by other depressive syndromes (7.3%, 95%CI 5.3–10.3), major depression (5.1%, 95%CI 3.2–7.7), and anxiety disorder (1.3%, 95%CI 0.4–2.9). PTSD prevalence was 2.8% (95%CI 1.2–4.3). Current smoking was reported by 20.7% (95% CI 16.8–25.5), any current alcohol intake by 1.8% (95% CI 0.7–3.5), and drug use by 0.9% (95% CI 0.2–2.9).

### Unadjusted associations with mental disorders

Unadjusted associations of socio-demographic factors with CMD and individual sub-categories are displayed in Table 2. CMD as a whole was associated with older age (i.e. displacement as an adult), female gender, divorced or widowed status, unemployment, lower education, and food insecurity. When analysed against CMD sub-categories, the associations with age and gender were primarily accounted for by somatoform disorder, whereas those with marital status, education and employment were also evident for depression, and that for food insecurity was more prominent for depression than somatoform disorder. For PTSD as an outcome, significant associations were only found with widowed/divorced marital status (OR 4.9, 95% CI 1.1–21.1).

**Table 2:** Unadjusted logistic regression analyses of associations between socio-demographic factors and CMD and its component disorders.

		CMD sub-group			
	Any CMD	Somatoform disorder	Major Depression	Other Depression	Anxiety
Characteristic	OR(95%CI)	OR (95%CI)	OR (95%CI)	OR (95%CI)	OR (95%CI)
**Age (years)**					
*18–21 (born after displacement)*	Reference	Reference	Reference	Reference	*
*22–37 (child at displacement)*	0.8 (0.2–2.7)	1.5 (0.3–6.7)	0.8 (0.1–7.5)	0.4 (0.1–2.2)	*
*38–65 (adult at displacement)*	**3.5 (1.2–10.3)**	**5.0 (1.1–21.3)**	3.4 (0.4–26.1)	2.7 (0.6–12.2)	*
**Gender**					
*Male*	Reference	Reference	Reference	Reference	Reference
*Female*	**1.6 (1.0–2.7)**	**2.0(1.1–3.8)**	1.1 (0.4–2.6)	1.1 (0.5–2.5)	0.6 (0.1–3.0)
**Marital status**					
*Married*	Reference	Reference	Reference	Reference	Reference
*Widowed/Divorced*	**4.9 (2.4–9.9)**	**2.2 (1.0–4.9)**	**7.0 (2.7–18.3)**	**5.0 (2.0–12.7)**	*
*Never married*	**0.4 (0.1–0.9)**	0.3 (0.1–1.1)	0.8 (0.2–3.5)	0.4 (0.1–1.8)	2.6 (0.5–14.6)
**Education**					
*Primary (Up to grade 5)*	**4.0 (1.5–10.1)**	**2.9 (1.1–8.2)**	6.3 (0.7–50.3)	**4.9 (1.0–22.1)**	0.5 (0.1–8.6)
*Secondary (Up to OL)*	1.7 (0.7–4.3)	1.6 (0.6–4.3)	2.5 (0.3–20.1)	1.9 (0.4–8.5)	0.9 (0.1–8.1)
*Post-secondary (AL & above)*	Reference	Reference	Reference	Reference	Reference
**Financial debt**					
*Indebted*	1.0 (0.6–1.6)	1.0 (0.6–1.7)	0.8 (0.3–2.0)	1.6 (0.8–3.3)	1.3 (0.2–6.4)
*No debts*	Reference	Reference	Reference	Reference	Reference
**Food security (last year)**					
*Sufficient food*	**1.7 (1.0–2.8)**	1.3 (0.7–2.2)	2.0 (0.8–4.6)	**3.4 (1.6–7.1)**	*
*Lack of sufficient food*	Reference	Reference	Reference	Reference	*
**Employment**					
*Employed*	Reference	Reference	Reference	Reference	Reference
*Unemployed*	**2.8 (1.6–4.9)**	**3.0 (1.6–5.7)**	2.0 (0.7–5.1)	2.3 (1.0–5.2)	3.5 (0.4–30.5)

* Insufficient cell sizes.

### Multivariable analysis of factors associated with mental disorders

Multivariable logistic regression analyses included two models of mutually adjusted associations of socio-demographic variables with CMD as summarised in table 3. In model 1, widowed/divorced marital status and food insecurity were significantly associated with any CMD, with employment additionally associated in model 2.

**Table 3:** Logistic regression analyses of associations between socio-demographic factors and CMD.

	Any CMD		
	Unadjusted	aModel 1 - Adjusted	bModel 2 - Adjusted
Characteristic	OR (95%CI)	OR (95%CI)	OR (95%CI)
**Age**			
*18–21 (born after displacement)*	Reference	Reference	Reference
*22–37 (child at displacement)*	0.8 (0.27–2.7)	0.6 (0.1–2.4)	0.6 (0.1–2.5)
*38–65 (adult at displacement)*	**3.5 (1.2–10.3)**	1.8 (0.4–7.7)	1.9 (0.5–8.0)
**Gender**			
*Male*	Reference	Reference	Reference
*Female*	**1.6 (1.0–2.7)**	1.5 (0.8–2.7)	0.5 (0.2–1.2)
**Marital status**			
*Married*	Reference	Reference	Reference
*Widowed/Divorced*	**4.9 (2.4–9.9)**	**3.0 (1.4–6.4)**	**3.4 (1.5–7.5)**
*Never married*	**0.4 (0.1–0.9)**	0.5 (0.1–1.9)	0.5 (0.1–1.8)
**Education**			
*Primary (Up to grade 5)*	**4.0 (1.5–10.1)**	1.3 (0.4–3.2)	1.1 (0.4–3.0)
*Secondary (Up to OL)*	1.7 (0.7–4.3)	1.5 (0.5–4.0)	1.1 (0.4–3.2)
*Post-secondary (AL & above)*	Reference	Reference	Reference
**Financial debt**			
*Indebted*	1.0 (0.6–1.6)	0.9 (0.5–1.5)	0.9 (0.5–1.5)
*No debts*	Reference	Reference	Reference
**Food security (last year)**			
*Sufficient food*	Reference	Reference	Reference
*Lack of sufficient food*	**1.7 (1.0–2.8)**	**1.6 (1.0–2.8)**	**1.7 (1.0–3.0)**
**Employment**			
*Employed*	Reference	-	Reference
*Unemployed*	**2.8 (1.6–4.9)**	-	**4.3 (1.8–10.3)**

a *Model 1 - Adjusted for all variable excluding employment.*

b *Model 2 - Adjusted for all variables including employment.*

*Note: McFadden's R^2^ for Model 1 = 0.1135, McFadden's R^2^ for Model 2 = 0.1421.*

## Discussion

This is the first study investigating mental health impact of forced and prolonged internal displacement in Sri Lanka and one of few such studies of prolonged internal displacement worldwide. Considering prevalences of mental disorder, the prevalences of somatoform disorder (14.0%) and major depression (5.1%) were considerably higher than national estimates (4.2% and 2.6% respectively) [35]. However, the prevalence of CMD appears to be relatively low in the Sri Lankan sample (18.8%) compared to that observed in other international studies of IDP populations (Colombia; 27.2%, Ethiopia; 27.8%, Palestine; 40.3%, Cambodia; 57.7%, Algeria; 62.3%) [19], [23]. PTSD prevalence (2.8%) in the COMRAID sample is also lower when compared to a previous Sri Lanka study (7.0%) and international data (Guatemala; 11.8%, Afghanistan; 20.4%) [22], [17]. This may be attributable to the longer duration of displacement and time since the traumatic experience of forced migration, as well as by the inclusion of generations born in displacement [15], [44]. In interpreting these findings, the living conditions in displacement, compared in context to many other developing countries affected by conflict (especially African countries) have to be carefully considered [3]. These IDPs did not face continuous conflict-related trauma after displacement as the resettlement area was not significantly affected by the conflict [34].

However, the current CMD prevalence (especially high levels of somatoform disorder) may well reflect experiences of living in prolonged displacement, and it is worth questioning the extent to which well-recognised economic challenges in these circumstances are compounded by a sense of being cut-off from original homes and livelihoods, especially among older IDPs [3], [5], [8]. Although unadjusted findings showed associations between being displaced as an adult and mental ill health, these associations were non-significant when considered with other covariates. The absence of a clear association between age and depression or anxiety specifically in COMRAID, in contrast to the more commonly found association may reflect lack of statistical power, but might also be due to cultural differences - for example, possibly a relative excess in those with younger migration ages, obscuring a positive association with current age. As adults, IDPs would have had firmly developed sense of belonging, established livelihoods and social structures in place. The sudden loss of all these and the ability to understand the trauma of forced displacement may predispose the adult IDPs to develop increased risk for mental illnesses [4], [16]. However, the finding that other factors became more salient after adjustment – for example, unemployment, food insecurity and divorced/widowed status – suggests that at least some of the age-related risk may be accounted for by accumulated post-migration stressors rather than the original migration itself.

A higher prevalence of mental disorders has been observed in women among other conflict-affected communities [8]. In COMRAID, this association was not observed independently for CMD as a whole although it persisted for somatoform disorder as a sub-group (data not shown). This lack of an association with CMD may reflect an increased susceptibility of women to present the impact of displacement trauma through somatic symptoms. Furthermore, although depression and anxiety are commonly observed to have a higher prevalence in women in other contexts, this was not observed in COMRAID. This finding might be attributed to a higher chance of males being exposed to traumatic events during displacement and/or the higher socio-economic burden placed on males during the post-displacement period to provide for families. However, further research is required for confirmation.

Widowed and/or divorced status was more independently associated with CMD as well as being more common in female participants (data not shown). The lack of a husband/partner, lack of livelihood, loss of social structure, gender-associated stressors linked to displacement event and other gender-specific factors may all contribute to the higher risk of mental disorder in women [20], [21].

Unemployment was significantly associated with mental disorders in the sample. It may have a mediating effect between several socio-economic indicators (low education, female gender and lack of food security) and mental ill health, explaining for example the marked difference between the odds ratios for gender and CMD in models 1 and 2 of Table 3. Unemployment has been frequently found to be associated with mental disorders in international research [45], including in refugee populations [46]. Lack of proper employment may be due to geographical resource limitations, as the IDP population has substantially grown in numbers since initial displacement. Displacement itself may also have acted as a barrier against obtaining sustainable employment, as many traditional employment pathways were disrupted [18]. Finally, difficulties in engaging host communities to accept IDP workers and tensions emanating from limited available resources may also compound unemployment and related socio-economic situation. Lack of food security was another important factor associated with mental ill health. Probably linked to unemployment, a large proportion of IDP households were found to lack sufficient food sources, adding extra stress and burden to the existing displaced situation. Lack of food security may play an important role with creating direct or indirect impact on mental health of the lives of IDPs [45].

PTSD was only significantly associated with widowed/divorced marital status in the COMRAID study (unadjusted OR 4.9, 95% CI 1.1–21.1). Two previous studies among IDPs in Northern Uganda and Southern Sudan had similarly shown that those who were widowed or divorced were more likely to develop PTSD symptoms [17], [21]. However, in the COMRAID sample, PTSD prevalence was relatively low (overall 2.8%) while the highest number of people with PTSD belonged to widowed/divorced category. These reasons may explain the lack of significant associations of socio-demographic factors with PTSD and the apparent strength of the association with widowed/divorced marital status.

A study conducted among IDPs in Darfur also found that older age, lack of employment and lack of food security were associated with mental disorder, as well as an association between female gender and somatic symptoms [47]. These findings are remarkably similar to COMRAID results, presenting important evidence that factors associated with mental disorders among IDPs might in fact be very similar across cultural, social and geographical boundaries. This evidence strengthens the existing need to address issues faced by these vulnerable populations through broader, multi-disciplinary approaches. Studies looking at broader mental health of populations affected by prolonged displacement, not limited to few selected disorders such as PTSD or depression are needed. Research priorities require refocusing on the broader spectrum of mental disorders, stepping out of the ‘trauma’ model, in order to develop effective and viable interventions for these populations across the world.

### Strengths and limitations

Key strengths of this study include the ethically and culturally sensitive method of approaching and interviewing members of this community resulting in the complete response rate [25]. Although the participant community was primarily a closed, religiously specific group, through establishing good rapport with religious leaders, community leaders and the community itself, the study team was able to gather valuable data and insights into the past experiences and current circumstances of these forced internal migrants. The recruited sample represents a true cross-section of the wider northern Muslim IDP community, and the results can be generalised within limits to other IDP populations in Sri Lanka, although most other such groups have experienced relatively short term (a few months or years) displacement. To our knowledge, this study is unique in its attempt to explore the potential impact of prolonged, forced internal displacement of 20 years (1990–2010) on mental health in those affected. Previous studies have provided only limited information on long-term displacement. Although there are likely to be variations in circumstances at an international level, it is also possible that commonalities exist across IDP populations so that these findings may be useful in managing other IDP populations in the world, especially those affected by prolonged, forced migration in developing countries. Considering limitations, the cross-sectional design of the study does not allow causal relationships to be inferred (for example, it is possible that mental disorder may influence employment status as well as *vice versa*). The sample size was chosen to allow the prevalence to be measured with sufficient accuracy and may not have been sufficient for some of the covariate analyses carried out. The Patient Health Questionnaire was chosen as it has been previously translated and used among Sri Lankan populations, although not specifically in this IDP group. Measures chosen for the analyses described here focused on demographic and socioeconomic status; other subsequent work will explore in more detail previous stressors and individual resilience, felt to be beyond the scope of this manuscript. Discrepancies arising due to the different instruments used to measure mental disorders in conflict settings have been previously documented [19]. The female preponderance in the sample was not due to sampling error because there were no missing or refusing participants, and therefore reflects the nature of the source population – partly survival bias in the context of the long-running conflict in Sri Lanka, and partly out-migration of men due to employment necessity. The IDP community is characterised by high fluidity of displacement status, especially after the end of conflict, which may also have affected the sampling.

This first comprehensive study exploring the prolonged forced displacement for over 20 years of this minority ethnic group in Sri Lanka found important associations between social, economic and demographic covariates and prevalence of common mental disorders and PTSD. Our work confirms evidence that has emerged from global IDP hotspots. However, due to the unique characteristics of the participant community, prolonged nature of displacement and the displacement ordeal, these findings add new insight to the impact of forced internal displacement on mental health globally.
